# Comparative Studies on Retroviral Proteases: Substrate Specificity

**DOI:** 10.3390/v2010147

**Published:** 2010-01-14

**Authors:** József Tözsér

**Affiliations:** Department of Biochemistry and Molecular Biology, Research Center for Molecular Medicine, Medical and Health Science Center, University of Debrecen, Egyetem tér 1, Debrecen, Hungary; E-Mail: tozser@med.unideb.hu; Tel.: +36-52-416-432; Fax: +36-52-314-989

**Keywords:** retroviral proteases, HIV protease, substrate specificity

## Abstract

Exogenous retroviruses are subclassified into seven *genera* and include viruses that cause diseases in humans. The viral Gag and Gag-Pro-Pol polyproteins are processed by the retroviral protease in the last stage of replication and inhibitors of the HIV-1 protease are widely used in AIDS therapy. Resistant mutations occur in response to the drug therapy introducing residues that are frequently found in the equivalent position of other retroviral proteases. Therefore, besides helping to understand the general and specific features of these enzymes, comparative studies of retroviral proteases may help to understand the mutational capacity of the HIV-1 protease.

## 1. Introduction

**Figure 1.**
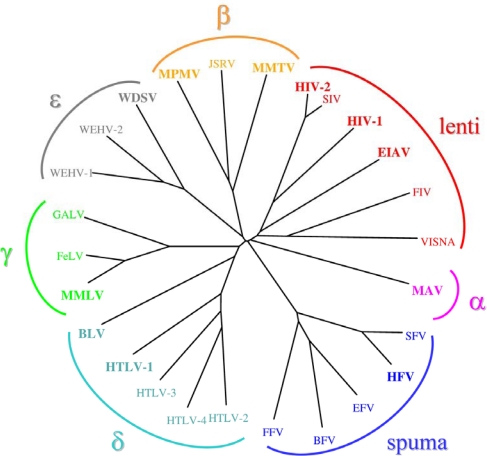
Phylogenetic relationship of retroviral proteases. Sequence alignment and the phylogenetic tree was made by ClustalW and Phylip programs, respectively. Abbreviations used: BLV, bovine leukemia virus; BFV, bovine foamy virus; EIAV, equine infectious anemia virus; EFV, equine foamy virus; FeLV, feline leukemia virus; FFV, feline foamy virus; FIV, feline immunodeficiency virus; GALV, gibbon-ape leukemia virus; HIV, human immunodeficiency virus; HTLV, human T-lymphotropic virus; HFV, human foamy virus; JSRV, jaagsiekte sheep retrovirus; MAV, myeloblastosis associated virus; MMLV, Moloney murine leukemia virus; MMTV, mouse mammary tumor virus; MPMV, Mason-Pfizer monkey virus; SIV, simian immunodeficiency virus; SFV, simian foamy virus; WDSV, walleye dermal sarcoma virus; WEHV, walleye epidermal hyperplasia virus; Proteases reviewed in this paper are in larger bold.

All replication competent retroviruses code for a protease (PR) that has an essential function in viral replication, therefore the PR of human retroviruses, especially that of human immunodeficiency virus type 1 (HIV-1), is a good and well-explored target for antiviral therapy. Currently retroviruses are classified into seven *genera*: the phylogenetic tree created for these viruses on the basis of the protease sequences is shown in Figure 1: this relationship is similar to that established based on the whole sequences [1].

**Figure 2.**
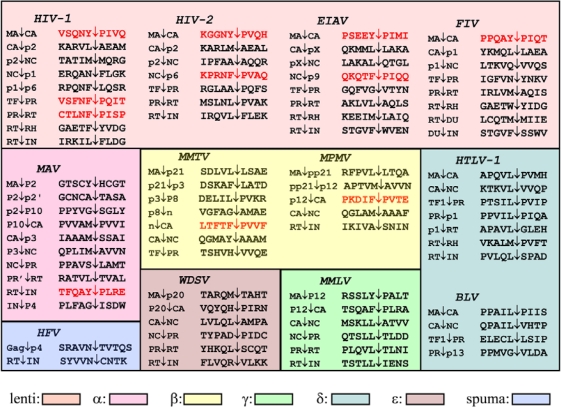
Naturally occurring cleavage sites in retroviral Gag and Gag-Pro-Pol polyproteins. The site cleaved by the cognate retroviral protease is indicated by an arrow. Type 1 cleavage sites are in red. Abbreviations used: CA, capsid; NC, nucleocapsid; TF, transframe protein; PR, protease; RH, RNaseH; IN, integrase; DU, dUTPase. Proteins and peptides with unidentified functions are abbreviated with the size of the protein in kDa (e.g., p12 is a protein having 12 kDa, while pp refers to phosphoprotein), as pX, or by the letter n.

The viral PR plays a critical role at the last stage of viral replication by processing of the Gag and Gag-Pro-Pol polyproteins at a limited number of sites (Figure 2), by converting the morphologically distinct “immature” virions into “mature” ones [2]. Based on the sites of processing, it is not possible to give a consensus substrate sequence (unlike e.g., for caspases), and this is a general characteristic of the retroviral proteases. Nevertheless, based on similarities in amino acid sequences, primate lentiviral (HIV-1, HIV-2, SIV) cleavage sites were grouped into three classes [3], while analysis of a broader range of retroviral protease cleavage site sequences suggested two types of cleavage sites [4]. Later systematic specificity studies on HIV-1 PR also verified the existence of two types of cleavage sites, type 1 - having an aromatic residue and Pro - (highlighted in red in Figure 2), and type 2 having hydrophobic residues (excluding Pro) at the site of cleavage [5,6], defined as P1 and P1’ positions [7], respectively. These classical cleavage type sites also showed different preferences for the P2 and P2′ positions [5,6].

The type 1 cleavage site was thought to be important in defining specificity. With the exception of pepsin, cellular proteases are not capable to efficiently process peptide bonds at the imino side of Pro residue. The unique nature of Pro at the P1’ position of retroviral cleavage sites was recognized even before the discovery of the PR and implied that these sites should be processed by a virally-coded enzyme [8]. It should be noted, that many of the cleavage sites do not fit into these classifications (e.g., they contain polar residue at P1 or P1’ or contain Pro after a nonaromatic residue, see Figure 2), therefore this classification might be an oversimplification.

**Figure 3.**
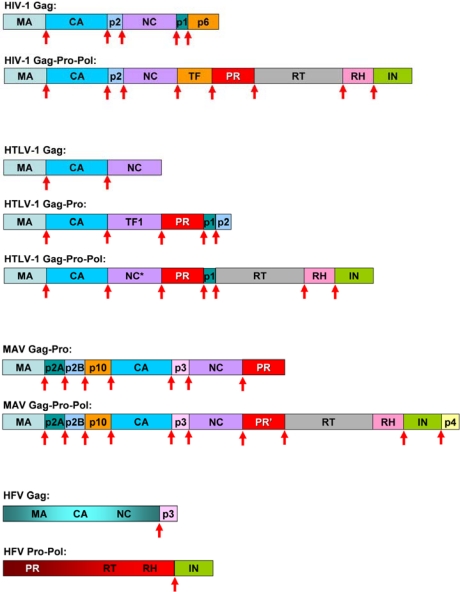
Organization of the Gag-Pro-Pol proteins in lentiviral HIV-1, deltaretroviral HTLV-1, alpharetroviral MAV and spumaretroviral HFV. Sites of PR processing are indicated by arrows.

The PR is coded in the so called *pro* gene, that is either in frame with the Gag and Pol (as in case of MAV) or is produced with a stop codon suppression (exemplified by MMLV), frameshift mechanism exemplified by HIV-1) or by a splicing event (as it is the case of the HFV PR). As examples, the organization of the Gag-Pro-Pol proteins of HIV-1 HTLV-1, MAV and HFV is provided in Figure 3.

**Figure 4.**
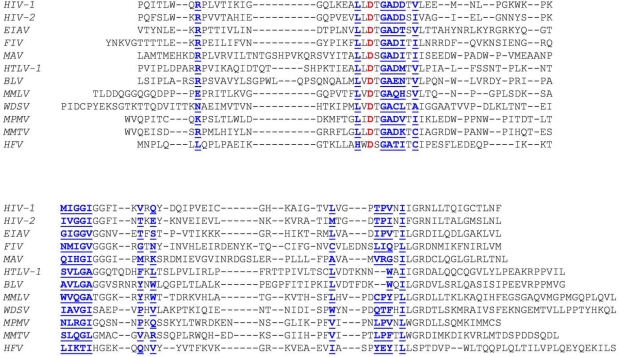
Structure-based sequence alignment of the retroviral proteases. Active site aspartate residues are in red, amino acid residues involved in substrate binding are in blue and underlined.

The way of PR synthesis appears to be in a good correlation with the activity of the enzymes: while proteases produced by frameshifting or stop codon suppression, and therefore being present only in 5–20% amount compared to the Gag typically have high specific activity, the protease of MAV produced in frame of Gag and therefore being in an equivalent amount with this substrate has a substantially lower specific activity (see below). The PR is an aspartyl protease, acts as a homodimer, as described in detail by Weber *et al*. in this issue [9]. Structure-based sequence comparison of the proteases reviewed in this paper is provided in Figure 4.

Specificity studies of retroviral proteases typically utilize protein substrates or oligopeptides. As crystal structures have been determined for various retroviral proteases [10] or molecular models could be built with high certainty, specificity studies have been frequently complemented with molecular modeling to understand the molecular basis of PR specificity. However, as no generally accepted and widely used standard assay conditions have been established in the retroviral protease field, and the PR is highly sensitive to the pH, ionic strength and type of the substrate (e.g., protein *versus* peptide, or the presence of ionizable side chains such as Glu), it is difficult to quantitatively compare data published by different laboratories. Furthermore, while it has been a commonly used strategy to compare the specificity of a PR of a given retrovirus to that of another one (typically to that of HIV-1), only a few studies dealt with the comparison of more enzymes. In this review, after the description of the specificity results regarding some representative proteases, the comparative studies involving a set of proteases will be summarized.

## 2. HIV-1 protease

**Figure 5.**
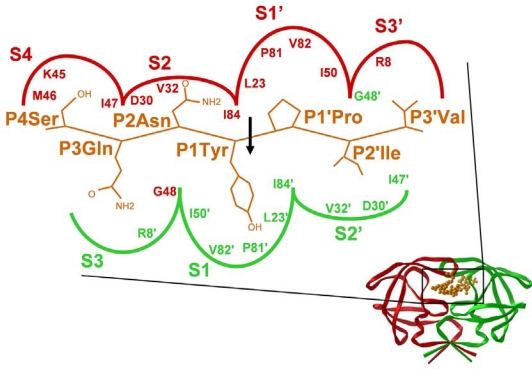
Schematic representation of the HIV-1 MA↓CA cleavage site substrate in the S4 S3′ subsites of HIV-1 PR. The indicated substrate sequence was modeled into the binding site of the crystallographic structure of the PR. The relative size of each subsite is indicated approximately by the area enclosed by the curved line.

By far the most of our knowledge on the specificity of retroviral proteases has been obtained by studying that of HIV-1 PR as described in several reviews [11–14]. In type 1 cleavage sites of primate lentiviruses (HIV-1, HIV-2 and SIV), there is a preference for Asn at P2 and beta-branched hydrophobic residue (Val or Ile) at P2’, while in type 2 cleavage sites the P2 residue is typically beta branched and the P2’ residue is Glu or Gln (Figure 2). Although only the CA↓p2 site of HIV-1 contains the charged Glu at P2’, this residue was suggested to play a regulatory role in the viral protein processing, as cleavage at this site is accelerated by lower pH [15]. A schematic diagram of the substrate binding site of HIV-1 PR with modeled interaction of the bound residues of the MA↓CA cleavage site is given in Figure 5.

The substrate binds to the enzyme in an extended beta conformation, and it is anchored by several hydrogen bonds: a very similar binding mode is observed with the inhibitors of the PR. The HIV-1 PR recognizes at least seven substrate residues, from P4 to P3’ and each amino acid side chain of the substrate fits in successive subsites (S4 to S3′) formed by PR residues (Figure 5). Although the HIV-1 PR (similarly to the proteases of other retroviruses) is a symmetrical dimer of two identical subunits, the residues of naturally occurring cleavage sites do not show symmetrical arrangements (Figure 2) and no obvious symmetrical substrate preference has been observed for the specificity of HIV-1 PR [6]. Nevertheless, modeling showed that the same residues of the two enzyme subunits interact with the appropriate substrate residues at both sides of the scissile bond (Figure 5). Based on detailed specificity studies as well as HIV-1 PR-inhibitor crystal structures, there appear to be a very strong sequence context dependence of the specificity of HIV-1 PR, that also provides an explanation for the lack of consensus sequences, and appears to be a general feature for the retroviral proteases. As the substrate binding pockets are overlapping, interactions could occur between the side chains of the substrate [6], and these interactions could lead to alternative positioning of a given side chain [16] causing an energetically non-additive nature of the ligand side chain-binding pocket interactions [14]. Crystallographic studies of active site mutant HIV-1 PR complexed with substrate peptides [17] and of active enzyme complexed with substrate analogs [18] suggested the molecular basis of this phenomenon. Unlike in the Gag and Gag-Pro-Pol cleavage sites, cellular protein cleavage sites frequently contain charged residues, especially Glu at P2’ [11]. The reason for the markedly different subsite preference in viral and cellular proteins of the HIV-1 PR is not known, but might at least partially be due to the different assay conditions applied. In spite of the strong sequence context-dependent nature of the HIV-1 PR specificity, the basic features of the substrate binding subsites could be determined independently of the substrates used. The S4 subsite is close to the protein surface and partly exposed to solvent, and the HIV-1 S4 binding site prefers small, even hydrophilic residues. The S3–S3’ region is generally hydrophobic, although the naturally occurring cleavage sites contain at least two polar (or even charged) residues at some of these positions (Figure 2). It should be taken into account, that these regions are expected to be linker sequences between protein domains of Gag(-Pro-Pol), a function that requires a certain degree of hydrophilic nature of the given sequence. Besides the hydrophobicity, the size of residues as well as the presence or absence of branching (especially beta branches) appear to be important in determining specificity. The S3 (like S3’) subsite is relatively large, and is partly exposed to solvent at the surface of the enzyme. The P3 side chain may be positioned either to interact with the more polar residues of the PR surface (as shown in Figure 5), or to interact with the hydrophobic internal residues of the enzyme: various residues are accepted at this position by HIV-1 PR. Studies with type 1 peptide substrates indicated a preference for small residues like Cys or Asn at P2 position and preference for beta-branched Val or Ile at P2′ position [19], while studies with type 2 peptides showed preference for beta-branched residues, especially for Val at P2, and Glu for P2′ [5], in good agreement with the type 1/type 2 cleavage site classification. Further studies revealed, that in case of tight packing of both S1 and S1′ with Tyr residues, a relatively larger residue at either P2 or P2′ is preferable, but not at both positions [6].

**Figure 6.**
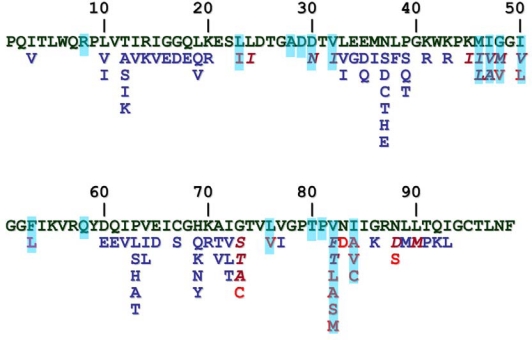
Sequence polymorphism and drug-resistant mutations of HIV-1 PR. Sequence of the HIV-1_HXB2_ PR is in green, natural variations are in blue, resistant mutations are in red. Those residues that can be observed at the equivalent position of another retroviral PR are shown in italics. Residues that are involved in ligand binding are marked with blue background.

As a consequence of the error-prone nature of the reverse transcriptase, HIV-1 (as typically other retroviruses) is a quasi-species, having natural sequence variations throughout its genome, including the *pro* gene (Figure 6). Furthermore, HIV-1 PR mutates extensively as a response of the selective pressure exerted by the PR inhibitors used in drug therapy. Unlike the natural variations, these mutations causing drug resistance tend to occur in the substrate binding region of the protease (reviewed in [20]) and frequently the resistant mutations introduce residues that can be found at the equivalent position of other retroviral proteases (Figure 6), therefore comparative study of retroviral proteases is a promising approach not only to recognize general and specific features of the PR, but also to discover the mutational capacity of the HIV-1 PR. Approximately half of the residues have been found to be mutated as a consequence of the protease inhibitor applications, and have been implicated in drug resistance. Structural analysis of mutant proteases are reviewed in this issue of *Viruses* [9]. As the drug-resistant mutations typically occur in the substrate binding region, they are expected to change the specificity of the enzyme, as verified by various studies, and reviewed in [21]. However, an important feature of the drug resistant mutants is that they are still capable to cleave the viral processing sites fairly efficiently. A property, termed vitality was introduced as a measure of the selective advantage of different mutants in the presence of a given inhibitor [22]. Crystallographic analysis of HIV-1 PR substrate and inhibitor complexes suggested that the inhibitor envelope (the space filled by the inhibitor molecule) protrudes from the substrate envelope, therefore the protease is capable to mutate interacting residues into smaller ones to substantially alter the inhibitor binding without dramatically affecting the substrate binding and hydrolysis [23].

## 3. HIV-2 protease

In comparison with HIV-1, HIV-2 is less pathogenic and less transmissible, with lower viral loads while asymptomatic and a slower progression to AIDS [24]. HIV-2 PR, like the HIV-1 enzyme, was able to cleave all oligopeptides representing the main Gag-Pro-Pol cleavage sites in HIV-1 and in HIV-2 [25], and similar result was also observed with *in vitro* polyprotein processing [26,27]. Although initial reports suggested substantial differences in the specificity of HIV proteases [28,29], subsequent studies suggested that the specificity of these two enzymes are rather similar [19,25,30] in good agreement with their very similar substrate binding sites: they differ in only four residues appearing as conservative substitutions (Val32Ile, Ile47Val, Leu76Met, Val82Ile) with the gain or loss of methyl groups. Specificity studies in combination with molecular modeling have revealed the importance of these residues to explain differences in substrate specificity between HIV-1 and HIV-2 proteases [19,30,31]. Although clinical inhibitors of HIV-1 PR typically less potent against HIV-2 PR [32], they can be utilized in anti-HIV-2 therapy, as reviewed recently with various other aspects of HIV-2 PR [33]. Another primate lentivirus for which the specificity has been studied is that of SIV. The specificity of the enzyme appeared to be identical to that of HIV-2, in good agreement with the high homology: the two enzymes have almost identical sequences [34]. Although the resistant mutations in HIV-2 PR are also expected to change the enzyme specificity, as it is the case with HIV-1 PR, this has not been studied so far.

## 4. Nonprimate lentiviral proteases

The most studied nonprimate lentiviral proteases are those of EIAV and FIV. Purified EIAV PR was able to process recombinant Gag protein producing products having similar sized to those found in virions [35,36,37]. The specificity of EIAV PR was extensively characterized using both type 1 [38] and type 2 [37] substrate sets. While the S1 preference appeared to be for large hydrophobic residues, the preference for the P2 and P2’ positions were not only different from those of HIV proteases but also differed in the two sets: for example medium sized residues were preferred in the type 1 series while larger residues in the type 2 one. The length of the substrate pocket of the protease appeared to be more extended than that of HIV proteases, and studies with naturally occurring cleavage sites also suggested different features of these enzymes [39]. FIV PR and HIV-1 PR are only 23% identical at the amino acid level, and their substrate specificity was found to be substantially distinct [40–43]. It is of interest to note, that while FIV PR did cleave the peptides representing its own cleavage sites, it did not cleave the peptide representing the MA↓CA cleavage site of HIV-1 [44], while not only EIAV but several other proteases were found to be able to cleave a peptide representing this site [45].

## 5. Alpharetroviral proteases

Avian retroviruses belonging to the alpharetrovirus *genus* served as model systems for retroviral studies, and many of the early specificity studies on retroviral proteases were done with alpharetroviral proteases (reviewed in [2]). The mostly studied Myeloblastosis associated virus (MAV, also referred as AMV) and Rous sarcoma virus (RSV) proteases differ only by two amino acid residues that are not involved in ligand recognition, therefore they will be referred in this review as MAV PR. Importantly, twenty years ago, MAV PR was the first retroviral enzyme for which the crystal structure was determined [46], opening the way of structure based specificity interpretations and drug design. MAV PR was a focus of intensive specificity and mutagenesis studies [47–51]. Among others, peptides representing MAV cleavage sites were also utilized to characterize the enzyme and to compare its specificity to that of HIV-1 [49,52,53]. With one exception, all peptides representing natural cleavage sites in MAV were hydrolyzed by the MAV PR at the expected site, but only half of them were substrates of the HIV-1 PR [53]. By comparing the amino acids at the P1-P1’ region of the naturally occurring cleavage sites, it is obvious that unlike most proteases, MAV PR seems to tolerate glycine or polar residues (Figure 2). The comparison of the MAV PR specificity to that of HIV-1 PR was performed using peptide sets based on the MAV NC↓p2 (type 2) cleavage site [50] and HIV-1 MA↓CA type 1 cleavage site [53]. These studies revealed common features of the proteases including their strong sequence context-dependent nature, an extended binding region and more hydrophobic S4 site for MAV, and the molecular basis of the specificity differences.

## 6. Betaretroviral proteases

Specificity studies have been performed on the proteases of MMTV and MPMV, the prototypical members of the betaretrovirus *genus*. As with many other viral proteases, synthetic peptides representing naturally occurring cleavage sites in MMTV and MPMV were found to be good substrates of the MMTV PR [54] and MPMV PR [55], respectively. In these experiments some peptides representing cleavage sites in other retroviruses were also tested, and based on the results, the specificity of the MPMV PR was suggested to be more closely related to that of MAV, compared to that of HIV-1 [55]. MPMV PR was found to be able to process the CA protein in the early phase of viral replication [56], and *in vitro* studies of HIV-1 CA with HIV-1 PR suggested that this protein is also substrate of the enzyme [56,57].

## 7. Gammaretroviral proteases

Similar to MAV, Moloney murine leukemia virus (MMLV) is one of the model retroviruses that was studied extensively even before the discovery of HIV, its protease was one of the first one to be isolated from virions [58]. In spite of the importance of MMLV as a model system, only a few reports have dealt with its specificity, as reviewed previously together with other characteristics of the enzyme [59]. The oligopeptides representing the MMLV cleavage sites were tested using MMLV PR, and were found to be hydrolyzed at the expected sites [60,61]. Only three of the seven MMLV-representing substrates were cleaved by the HIV-1 PR, but one of these were cleaved at a nonauthentical site, while with one exception, MMLV PR was able to properly process the HIV-1-representing cleavage sites, although with lower rates [61]. Many of the viral processing sites cleaved by MMLV PR have Leu(Val,Ala)-Leu at the P2 and P1 positions, and these were relatively good substrates for the MMLV PR, but not hydrolyzed by HIV-1 PR. Besides the ability to cleave most of the HIV-1 cleavage sites, MMLV PR was also able to cleave several other oligopeptides, representing naturally occurring cleavage sites of various retroviruses [60] indicating a fairly broad specificity of this protease. In comparison with HIV-1 PR, the P4 and P2 positions appeared to be important in defining the specificity differences of the two enzymes using an HIV-1 MA↓CA site-based peptide series: branched-chain amino acid residues (Val, Ile, Leu) were the preferred ones at P4 and P2 by MMLV PR [62], while HIV-1 and HIV-2 proteases preferred rather smaller, more hydrophilic residues at these positions [19,62].

Recombinant, expressed proteins containing the MMLV cleavage sites were also used to study the specificity of MMLV (and HIV-1) proteases, and suggested that the most efficiently cleaved site is the CA↓NC one [61], in good agreement with peptide hydrolysis data [60] and with studies on bacterially expressed Gag-protease fusion proteins [63].

## 8. Deltaretroviral proteases

The protease of HTLV-1 and bovine leukemia virus (BLV) has been characterized among the members of the deltaretrovirus *genus*. HTLV-1, the first human retrovirus to be discovered, has been etiologically associated with a variety of diseases [64]. Cleavage of oligopeptide substrates representing naturally occurring cleavage sites in various retroviruses suggested that the specificity of the HTLV-1 PR is very close to that of BLV, but distinct from those of HIV-1 and MAV proteases [65]. In a more extended study, the majority (80%) of the peptides representing retroviral cleavage sites were hydrolyzed by both the BLV and HIV-1 proteases [66], but only half of them were processed by the HTLV-1 PR [67]. In another study, except one BLV peptide, which was hydrolyzed only by the BLV PR, all other studied HTLV-1 and BLV cleavage site-representing substrates were cleaved by both enzymes at the expected site, although in some cases with substantially different rates [68]. To characterize the specificity of the HTLV-1 PR in detail, a series of oligopeptides based on the MA↓CA cleavage site of HIV-1 was first utilized, but most of the substituted peptides were not hydrolyzed by the enzyme [67]. Therefore another peptide series based on the CA↓NC cleavage site of HTLV-1 was utilized to characterize the specificity of the PR of HTLV-1 [67] and later that of BLV [66] in comparison with that of HIV-1 PR. In these studies the substrate binding site of the HTLV-1 PR appeared to be more extended than that of the other two proteases. BLV PR showed a broader specificity compared to HTLV-1 PR and although both the BLV and HTLV proteases showed a preference for larger hydrophobic P2 and P1 residues, the BLV PR tolerated hydrophilic or even charged residues at these positions much better. Nevertheless, in most aspects, the specificity of individual subsites of the BLV PR resembled that of the HTLV-1 PR more closely, in good agreement with the more similar sets of residues predicted to be involved in substrate binding, compared with those of the HIV-1 PR [66].

## 9. Spumaretroviral proteases

Spumaretroviruses (foamy viruses) and their proteases have several unusual features: the Pol protein is synthesized independently from Gag using a splicing mechanism, viral particles contain almost full length reverse-transcribed linear cDNA, and the nucleocapsid domain does not have the consensus zinc finger motif [69], therefore they were grouped separately from the other retroviruses, collectively belonging to the orthoretroviruses [70]. In contrast to the other retroviruses, the foamy virus Gag and Pro-Pol do not appear to be efficiently processed, except at one site close to the C-terminus of Gag and between the RT↓IN of Pro-Pol (Figure 3) [71,72]. Although it was found to be essential for virus replication [73], the obtained catalytic constants for HFV PR were much lower than those previously determined for various mammalian retroviral proteases coded in fusion with the *pol* genes [74], but similar to those obtained previously with avian retrovirus PR produced equimolarly with the Gag [53]. The HFV cleavage site sequences substantially differ from those of other retroviruses (Figure 2) and they rather resemble the cleavage sites of retrotransposones of yeasts: in line with these similarities, HFV PR was able to hydrolyze some Ty1 and Ty3 site-representing peptides [74]. HFV PR was also able to hydrolyze some of the P2 substituted HIV-1 MA↓CA cleavage site-representing peptides [45] but not the unsubstituted one and the other substituted versions [75]. As HFV PR showed similar activities on substrates containing Ala, Val, or Cys at the P2 site but did not hydrolyze peptides with larger P2 residues, the S2 site of this enzyme appeared to be one of the smallest ones among the studied 11 retroviral proteases [45].

## 10. Epsilon retroviral proteases

Together with two other retroviruses infecting walleys, walley epidermal hyperplasia virus type 1 (WEHV-1) and type 2 (WEHV-2) and with the snakehead retrovirus (SNRV), walleye dermal sarcoma virus (WDSV) belong to the epsilonretroviruses. WDSV is associated with skin tumors in walleyes and it is the prototype of the epsilonretrovirus *genus* [76]. WDSV cleavage sites were inferred from the N-terminal sequence data on purified viral proteins [77]. WDSV PR has been cloned and characterized, while its specificity was inferred from the sequences of the naturally occurring cleavage sites and from sequence homology: the Gag and Pol cleavage sites contain Gln at P2 and the specificity of the enzyme was expected to show the highest similarity to those of gammaretroviruses [76]. Structural features of WDSV such as a single Cys-His box in NC of Gag and predicted stop codon suppression for translation of the Gag-Pro-Pol polyprotein are also similar to those of gammaretroviruses. Nevertheless, using the HIV-1 MA↓CA cleavage site-representing peptides the specificity of WDSV PR appeared to be substantially different from that of MMLV [45,75].

## 11. Comparative studies of retroviral proteases

**Figure 7.**
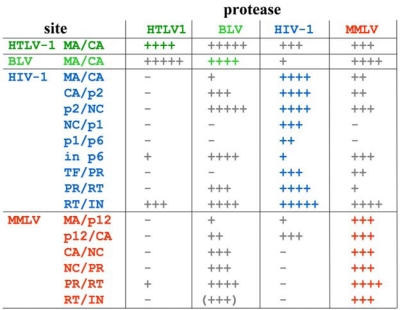
Cross-activity of retroviral proteases assayed on oligopeptide substrates representing naturally occurring cleavage sites. For simplicity, the k_cat_/K_m_ values (mM^−1^s^−1^) were indicated as follows: < 0. 1: +; 0.1 – 1: ++; 1–10: +++; 10–100: ++++; > 100: +++++. Parenthesis indicates shift in the site of cleavage.

As discussed above, most PR specificity studies dealt with the comparison of a given enzyme with HIV-1 (or HIV-2) protease. Only a few studies compared the specificity of a set of proteases. Particle associated retroviral proteins were assayed by homologous and heterologous PRs: MAV PR yielded efficient cleavage of the HIV-1 polyprotein but only at relatively high concentration. MPMV PR cleaved HIV-1 Gag very poorly, while BLV PR processed it efficiently but in altered sites [78]. In another *in vitro* cross-reactivity study on HIV-1, HIV-2, EIAV, HTLV-1 and HFV, the lentiviral proteases cleaved only the lentiviral Gag proteins [36]. Naturally occurring cleavage site peptides have also been cross-studied with proteases in many studies as described above. When data measured in identical conditions are compared [61,66,67], some enzymes (like HIV-1, MMLV, BLV PRs) appear to have a much broader specificity then HTLV-1 PR (Figure 7).

Interestingly, the specificity constant for some of these heterologous cleavages even succeded those obtained with the authentic cleavage sites. Shift in the site of cleavage was only observed very rarely, as indicated here for the BLV PR cleavage of an MMLV cleavage site. These results indicated, that the retroviral proteases retained a common core specificity. Nonviral proteins were also utilized to compare the specificity of proteases. Vimentin was tested as substrate of HIV-1, HIV-2, BLV, MPMV and MAV proteases, and found to be cleaved by them with different rates and sites [79]. Poly(A)-binding protein was also tested as substrate and was found to be processed by the PR of only MMTV, HIV-1 and HIV-2 but not by the PR of MMLV, HTLV-1 and SIV [80].

A large peptide series containing amino acid substituted versions of the HIV-1 MA↓CA cleavage site was utilized to characterize the S4-S1 binding sites of 11 proteases of retroviruses representing each of the seven *genera* of the family *Retroviridae* [45,75]. The S1 specificity of HIV-1, HIV-2, EIAV, AMV, MMLV and WDSV proteases appeared to be similar, showing the highest preference for Phe followed by Tyr, although the tolerance for smaller residues substantially differed. On the other hand, the PR of BLV showed the highest preference for Leu side chain at this position. The S1 subsite of retroviral proteases in general is large, hydrophobic and is well conserved [75]. Unlike the S1, the S2 subsite was found to be one of the most important ones for determining the substrate specificity differences of retroviral proteases. The P2 Asn preference of HIV-1 PR appeared to be exceptional, and a subgroup of proteases preferred small hydrophobic side chains, while the PRs of another group showed preference for larger hydrophobic residues (Ile and Leu) [45]. The S2 binding sites of retroviral proteases are relatively smaller, sterically restricted, mostly hydrophobic pockets where size complementarities appear to be the main specificity-determining features. In most cases the P2 residues of naturally occurring type 1 cleavage site sequences of the studied proteases agreed well with the observed P2 preferences [45]. Unlike with the internal binding sites, various residues were preferred when the S3 binding sites were mapped. Lentiviral proteases preferred the original Gln together with larger hydrophobic P3 residues. More pronounced preference for the large hydrophobic residues appeared to be characteristic for AMV, MPMV and MMTV proteases. On the other hand, smaller or polar, even charged residues are preferred by the MMLV, BLV and WDSV proteases [75]. The size of the residue appears to be the main specificity determinant at this position. Similar to S3, various residues were found to fit preferably to the S4 sites of the PRs [75]. It should be noted that this subsite is at the PR surface, and lacks the well-defined pocket of the internal binding sites.

It is of interest to know that while the cross-specificity is common in retroviral proteases, as demonstrated in Figure 7, HIV-1 clinical inhibitors typically only weakly inhibit, if at all, the other retroviral proteases [61,66]. So there is an apparent contradiction between the somewhat conserved specificity of these enzymes and the almost complete lack of inhibition by inhibitors specifically designed against HIV-1 PR. This contradiction might be due to the fact that the clinical inhibitors are typically rigid molecules, while substrates are more flexible, and capable of adapting to altered substrate binding sites in different retroviral PRs. The same phenomenon appears to be critical in drug resistant HIV-1 PR variants [17,18]. The development of resistance towards the drugs designed against HIV-1 PR is one of the main problems in the protease inhibitor therapy of AIDS. As discussed above, many of the mutations occurring in drug resistance introduce amino acids that can be found at the equivalent position in other retroviral proteases. Therefore, understanding the specificity similarities and differences of these enzymes may help to understand the mutational capacity of the HIV-1 protease, and to design broad-spectrum inhibitors against HIV-1 PR.
